# Validation of the Traumatic Antecedents Questionnaire using item response theory

**DOI:** 10.1002/brb3.1870

**Published:** 2020-10-01

**Authors:** Kyumin Park, Geumsook Shim, Bumseok Jeong

**Affiliations:** ^1^ Graduate School of Medical Science and Engineering Korea Advanced Institute for Science and Technology (KAIST) Daejeon Korea; ^2^ KAIST Clinic Pappalardo Center Korea Advanced Institute of Science and Technology (KAIST) Daejeon Korea; ^3^ KAIST Institute for Health Science and Technology Korea Advanced Institute of Science and Technology (KAIST) Daejeon Korea

**Keywords:** adolescent, child abuse, principal component analysis, psychometrics, surveys and questionnaires

## Abstract

**Background:**

Traumatic Antecedents Questionnaire (TAQ) is a traumatic experience scale that measures exposure to traumatic events across four age periods. Although the TAQ has good convergent validity with other traumatic scales, the classification of the domains and the psychometric properties of the scale has not been verified.

**Methods:**

A total of 290 young adults completed the TAQ, and 156 participated in the retest. The number of trauma domains was determined using principal component analysis. Rasch model was used for verifying the items that each domain might represent in one common measurement.

**Results:**

When scores were transformed as binary a code 0 and 1 from the original 4 categories, 8 domains were established consisting of Domestic violence, Sexual/other rare trauma, Incompetence, Caring family, Accidents to close person, Unstable caring environment, Safe environment, and Lack of sexual/extreme trauma. Most domains had acceptable psychometric properties with a mean‐square fit value within the range of 0.7–1.3. The Bland and Altman analysis suggest 98.7% of difference scores between test and retest were within ±2 standard deviations from the mean. TAQ severity showed a significant relationship with the multiplicity score of the Maltreatment and Abuse Chronology of Exposure scale (*r* = 0.677).

**Conclusions:**

The newly proposed scoring system and 8 domains for the TAQ demonstrated excellent internal consistency, test–retest reliability, and validity. Further studies are needed to develop new items in domains with less than 5 items to improve the psychometric properties of the scale and to create a maltreatment domain that includes bullying items.

## Graphical abstract

Through factor analysis, items of the TAQ were grouped into relevant domains, and the uni‐dimensionality of each domain was verified using IRT. When the Rasch model, the simplest form of IRT, is used to evaluate the difficulty distribution of items in the same scale, the probability of success for a person that an item is endorsed reflects the degree of the trauma experience and the difficulty of the item; in this case, the exposure level of the participant and the severity of the item.

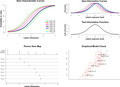

## 1 INTRODUCTION

Trauma could be considered as the origin of various psychiatric disorders (Martins et al., 2011) and has been associated with psychopathology (Yun et al., 2019). Traumatic experiences during development can affect function as well as the structure of brain in of healthy adolescents (Kim et al., 2019; Lee et al., 2015, 2017,2018) or young adults (Choi et al., 2009, 2012). The impact on psychiatric vulnerability and brain development is different depending on the age trauma is experienced (Choi et al., 2012). Therefore, determining the severity, extent, and when traumatic events were experienced is extremely important.

Traumatic experiences include abuse (emotional, verbal, sexual), neglect (physical, emotional), maltreatment/bullying, and witnessing violence. The Maltreatment and Abuse Chronology of Exposure (MACE) scale is has been used to retrospectively assess different forms of traumatic experiences (Teicher & Parigger, 2015). However, to assess traumatic experiences within the general population, a possible caveat of the 52‐item MACE is that participants have to respond to each item for each year between the ages of 1 to 18. Although the MACE scale has excellent test–retest reliability (Teicher & Parigger, 2015), an assessment of age periods, defined as a range or intervals, could lead to a less time‐consuming measurement.

The Traumatic Antecedents Questionnaire (TAQ) is a useful scale to detect past traumatic experiences (Luxenberg et al., 2001). The original 42‐item self‐report measure was developed by van der Kolk (B.A. van der Kolk, M.D., unpublished instrument, 1992). All items require a response for 4 ages, unlike MACE's 18. It suggests that TAQ is a less time‐consuming measurement and can be an alternative of MACE. Participants are asked to rate the extent to which each statement describes the experience on a scale from 0 to 3 or selecting “Don't know”. The response rating “0” means “never or not at all,” while “3” means “often or very much.” The Cronbach's α value for 37 items of unvalidated Korean version TAQ was reported as 0.88 (Kim et al., 2011).

Although the TAQ developed based on the Classical Test Theory has good convergent validity with other traumatic scales, the classification of the domains and the psychometric properties of the scale has not been verified. The confirmatory factor analysis (CFA) reproducing the covariance among items explains the differences between the observed score and the person's true score. However, this method does not analyze the specific properties of test items (e.g., how difficult each item is in the same domain). Thus, it is possible that very similar items, in terms of item difficulty, can be included in a specific domain of the scale. Recently, Item Response Theory (IRT) has been used to develop and verify the validation of psychometric tests based on responses and test taker characteristics. If a domain consisted only of items that are too general or specific, it could provide limited information for individuals at the opposite end of the severity spectrum. Furthermore, IRT models the probability of responses (e.g., four categories from “never or not at all” to “often or very much” in TAQ) to a particular item response based on a nonlinear model. Thus, IRT can test the validity of the response ratings for four categories of TAQ which rely on subjective memory or perceived feelings of traumatic experiences. Although this method provides a more specific representation of each item and domain, there is currently no study that evaluated the measurement properties of the TAQ based on IRT.

The aim of the present study was not to develop new items but rather to confirm that the existing TAQ items are valid in the same domain. Through principle component analysis, items of the TAQ were grouped into relevant domains, and the uni‐dimensionality of each domain was verified using IRT. When the Rasch model, the simplest form of IRT, is used to evaluate the difficulty distribution of items in the same scale, the probability of success for a person that an item is endorsed reflects the degree of the trauma experience and the difficulty of the item; in this case, the exposure level of the participant and the severity of the item.

## 2 MATERIALS AND METHODS

### 2.1 Ethics statement

This project was reviewed and approved by the Korea Advanced Institute of Science and Technology (KAIST) Institutional Review Board, Assurance # KH2017‐55.

### 2.2 Sample and data source

The TAQ Survey was completed by 290 undergraduate and graduate students (141 Female/149 Male, between 18 and 31 years of age (see S1 file, S1. Distribution of age and S2. Frequency of responses in each item)) at 10 Korean universities. All responses were provided in a Web‐based survey. All participants provided their consent and were screened for age, medicated state, education status, education level of parents, and familial financial status during the participant's childhood. Participants also completed the following questionnaires: Maltreatment and Abuse Chronology of Exposure Scale (MACE) (Teicher & Parigger, 2015), Verbal Abuse Questionnaire (VAQ) (Jeong et al., 2015), Verbal Affection Questionnaire (Polcari & Teicher, 2007), State‐Trait Anxiety Inventory (STAI) X1 and X2 (Spilberger, 1983), Center for Epidemiologic Studies Depression Scale (CES‐D)(Radloff, 1991), and the Limbic system checklist‐33 (Teicher et al., 1993). Among the 290 participants, 156 students (93 Female/63 Male) participated in the retest period 24–71 days (mean 32.3 days) postbaseline assessment.

### 2.3 Principle component analysis and item response theory

In this survey, the original version of the TAQ (42 items) per each of the four age periods, young childhood (birth to 6 years), school‐aged childhood (7–12), adolescence (13–18), and adulthood, were collected. However, 2 items of Family secrets (item number 9 and 24 in original TAQ) were deleted as their ceiling effects indicating problems with content validity in the recent version of the TAQ, we used the remaining 40 items for our analysis. Parallel analysis was performed using paramap package (1.9.1) to determine the number of components to retain from the principle component analysis (PCA). As too many items in one component will lose their validity as a set of items, parallel analysis was performed with the 4‐way scoring system in total. The detailed method is described with result in result 3.1.

In a Rasch model, item difficulties, δ, indicate the variation of how difficult a person experiences a specific item. A person's ability, θ, indicates probabilities of an experience for a person. For example, item A has a higher probability that people would experience that item compared with item B. If a person has theta of 0, his or her probability of the experience for item A is higher than 0.5 while the probability for item B is lower than 0.5. In the dichotomous case, 0 or 1, the probability of 1 is as follows:

$$p=P\left(X=1\right)=\frac{e^{\theta ‐\delta }}{1+e^{\theta ‐\delta }}$$

Logit function of *p*/(1 − *p*) is the difference between θ and δ.

$$\ln\left(\frac{p}{1‐p}\right)=\theta ‐\delta$$

Thus, differences in a person's ability between participant A and B can be calculated with (θA‐θB) (Wu & Adams, 2007).

### 2.4 Evaluation for item inclusion in a domain

Each component that resulted from PCA represents domains to assess exposure to the specific types of trauma during three developmental stages. A simple Rasch Model was used for item evaluation in each domain. In this model, both the exposure level of the participant and the severity of the given item were provided a logit score at the latent trait level instead of just a 0 or 1 value. This model shows that items can measure a range of exposure levels in a specific domain. More specifically, the item characteristic curves (ICC) illustrate the relationship between the severity of each item and the probability that the item will be endorsed by individuals at different exposure levels. Moreover, the item information curves (IIC) show how informative each item is in delineating a range of exposure levels (Bond & Fox, 2001; Mair & Hatzinger, 2007). Finally, the test information function indicates how informative items that constitute the domain are in participants with different latent exposure levels.

Rasch modeling software in R (eRm libraries version 1.0‐0) was used for calculations and plots. The mean‐square fit criteria were primarily used to evaluate the appropriateness of each item in a certain domain. The mean‐square fit is an index of fit of an item to the Rasch model. Both infit and outfit mean‐square fit statistics were determined by averaging the squared residual for each person and item combination. Anderson Likelihood Ratio test was performed to evaluate whether the item parameter estimate is invariable in a subgroup of the population. In the present study, participants were divided based on the median age of 23. Generalized linear mixed effect models were performed to investigate both developmental stage and gender effects (see S1 file ‐ S4 section and Table S9 for methods and results in detail).

### 2.5 Assessment of reliability and validity

#### 2.5.1 Reliability

Using the data from 156 participants who completed the repeat assessments, the test–retest reliability was calculated for both the overall TAQ score and each domain in each developmental period using Spearman's rank correlation coefficient and the Bland and Altman method. In this study, a certain item or domain was deemed reliable when the mean difference between test 1 and test 2 was not significantly different than zero and also when 95% of the differences between test 1 and test 2 fell within 2 standard deviations of the mean difference score. This is the definition of reliability adopted by the British Standards Institution (BSI, 1979). Cronbach's alpha value is a measure of the consistency of the test questions and measures the reliability of the items. Therefore, Cronbach's alpha was computed item consistency.

#### 2.5.2 Validity

Convergent validity was verified by evaluating the relationship of the TAQ score with other abuse scales such as the MACE, Verbal abuse Questionnaire, and Verbal affection questionnaire scores. The criteria for convergent validity were a correlation coefficient in the range of 0.6–0.8, which was used in a previous MACE study (Teicher & Parigger, 2015).

### 2.6 The relationship with other psychiatric symptom scores

The TAQ evaluates the extent of positive and negative experiences during young childhood, school‐aged childhood, and adolescence. That means it could potentially predict later developmental psychopathology. The STAI, CES‐D, and Limbic System Checklist‐33 were used to rate psychiatric symptom severity. The STAI and CES‐D were used to rate anxiety and depression, respectively, and Limbic System Checklist‐33 was used to assess limbic irritability which is known to be correlated with childhood abuse.

## 3 RESULTS

### 3.1 Classification of TAQ items and verification of new domains

**FIGURE 1:**
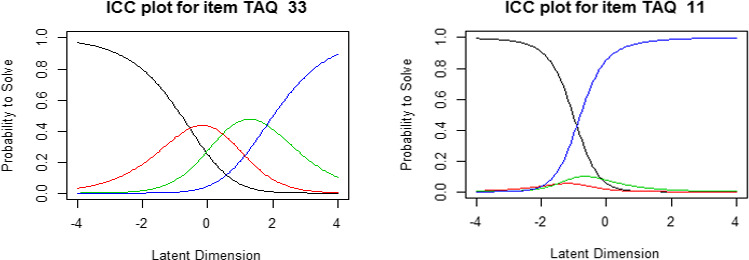
Good (left) and ill (right) fitted examples using the original 4 categories scoring

In the original TAQ scale scoring system, 3 categories were created by grouping “0 and 1” together as 0 and retaining “2 and 3” as independent categories. With the 3 categories, the number of factors based on the parallel analysis revealed 5 components. However, it seems that each component did not consist of relevant items. For example, TAQ item 28 “I abused alcohol and/or drugs” and item 36 “I saw sexual things that scared me” do not appear to share a similar nuance to be categorized in the same domain (S2 file). This is likely because too many items in one component result in the loss of their validity as a set of items. Further, PCA results demonstrated that relevant item construction was not found with binary categories when scores were transformed from “2” and “3” to “1” (S3 file) or 4 category scoring where “0,” “1,” “2,” and “3” were each an independent category (S4 file). Moreover, subsequent Rasch modeling with 4 category scoring showed ill‐fitted results. Most of the logit scores of the 4 categories scoring analysis were generally not evenly distributed or well separated (see Figure 1—Item characteristic curve (ICC) plot for TAQ 11). Specifically, in Figure 1, the 4 categories scoring is reasonable for item TAQ 33 but not in item TAQ 11. Both red and green colored curves give very little information compared to the other 2 in item TAQ 11, indicating that other scorings like 2 than 4 are better. Thus, to increase the sensitivity in the development and validation of the questionnaire by grouping items effectively with common components, we transformed $\left(0\to 0\right)$ and $\left(1,2,3\to 1\right)$.

Based on the PCA, we classified TAQ items into 8 new domains. These novel domains were named Domestic violence, Sexual/other rare trauma, Incompetence, Caring family, Accidents to close person, Unstable caring environment, Safe environment, Lack of sexual/extreme trauma.

Mean‐square fit values give an indication of the amount of randomness present in a measurement system. The values provide insight into the implication for measurement with values between 0.5 and 1.5 indicative of the system being “productive for measurement” (Linacre et al., 2002). The range of 0.7–1.3 is suggested for an acceptable fit. As presented in Table 1, none of the items had a fit statistic exceeding 1.5. This means that there were no items that did not contribute to the same underlying construct as the other items on the same scale. In the “Caring family” domain, the lowest MSQ value was out of the acceptable range. More specifically, item 29 in “Caring family” and item 16 in “Accidents to close person” could be considered to fit outside of the acceptable Outfit MSQ value. The *p* value of the Wald test of item 30 in “Domestic violence” and items 02, 04, and 08 in the “Incompetence” domains were below 0.05. However, since we were not in the process of selecting items and merely verifying the scale validity and reliability, we made an exception and included these items. Andersen's Likelihood Ratio (LR) Test showed that the LR value of the “Incompetence” domain significantly differed across age. This means that individuals at the same level of an underlying trait in “Incompetence” domain may respond differently to a specific item depending on age (aged 23 or above vs. below). In general, the acceptable standard of Cronbach's alpha value is 0.70 and a value of 0.80 or greater is preferred. Only the “Domestic Violence” domain had a Cronbach's alpha value greater than 0.7.

**TABLE 1:** Confirmation of internal consistency

Domain (number of items)	Mean‐square fit *t*‐value		Andersen test	Cronbach's alpha
	Outfit	Infit	LR value	
Domestic violence (7)	0.796–1.202	0.852–1.154	8.059	0.837
Sexual/other rare trauma (7)	0.697–1.029	0.736–1.037	8.044	0.579
Incompetence (7)	0.762–1.164	0.848–0.985	25.986*	0.639
Caring family (5)	0.343–0.961	0.554–0.913	5.929	0.618
Accidents to close person (3)	0.631–0.947	0.700–0.956	2.655	0.514
Unstable caring environment (4)	0.800–1.062	0.829–1.052	4.054	0.516
Safe environment (4)	0.973–1.006	0.871–1.010	2.884	0.582
Lack of sexual/extreme trauma (3)	0.918–1.071	0.925–1.067	2.240	0.458

Abbreviation: LR, Likelihood Ratio.

* *p* < .001.

Due to insufficient item parameters, 4 domains had 4 or fewer items which could not be scored for severity of exposure to the latent category by determining the number of items with positive endorsements and model parameters. Thus, we adopted the scoring method used in the MACE scale (Teicher & Parigger, 2015). The total number of items scored as “1” was normalized into 0–100 in each component. For example, if 5 items were scored as “1” among 7 items in a component, the normalized score of the component is 70 (5*14). This score was used to assess convergent validity.

**FIGURE 2:**
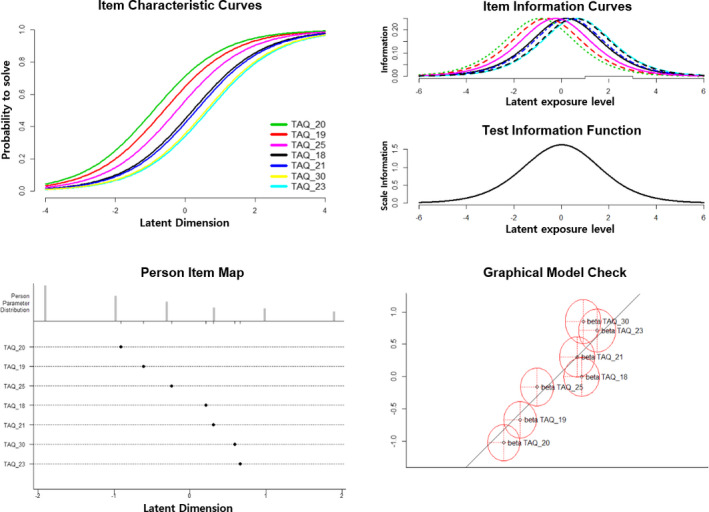
Results of the Rasch analysis for the Domestic violence domain

Figure 2 showed an example of good‐fitting IRT results of the 2 categories scoring. This domain of domestic violence was most informative with logit scores around 0 which suggests an average latent trauma level (see IIC plot in Figure 2). It covered the latent trait from logit scores of −2.89 (the lowest person value) to 2.87 (the highest person value) (see S3.1 section in supplemental document and Test Information Function plot in Figure 2). All curves cover a range of logit scores and were well distributed across the latent logit space (see ICC plot in Figure 2). The results suggest that scoring only “0” with “0” and “1” for the rest is more appropriate than others. Detail results for each domain are provided in the supplementary file (see S3. Results of Rasch analyses of each domain section in S1 file).

### 3.2 Test–retest reliability

**FIGURE 3:**
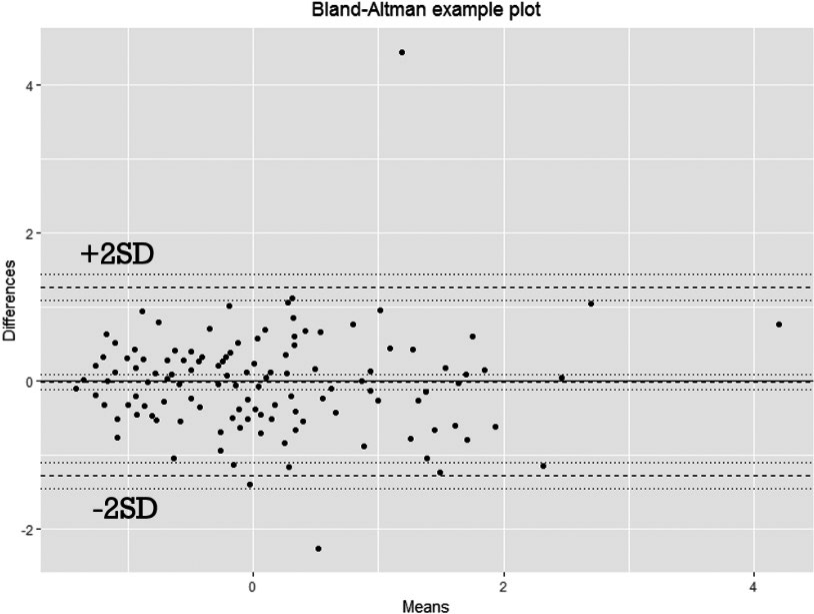
Results of the Bland and Altman analysis

Among the 290 participants, 156 students (93 Female/63 Male) participated in the retest. Total TAQ scores had a normal distribution (Wilcoxon signed‐rank value = 0.848, *p* < .05), and the Bland and Altman analysis showed 98.7% of difference scores were within ±2 *SD* of the mean difference score (Figure 3). Total TAQ scores between the two time points showed significant relationship (*r* = 0.798, *p* values <.05). Positive correlation coefficient values of test and retest reliability were found across each domain and also the 3 age periods.

Test–retest reliability for the overall degree of exposure to each type of maltreatment ranged from good to very good (defined as 0.5 < *r* < 0.8) for all of the domains (Table 2). Similarly, test–retest reliability for recollected scores at each age period showed good to very good reliability. The Spearman rank correlation coefficient of the period of maltreatment for the age period 0–6 years was 0.651, for 7–12 years was 0.698, and for 13–18 years was 0.720.

**TABLE 2:** Test–retest reliability by components

Type of maltreatment	Test–retest *r*
Domestic violence	0.768
Sexual/other rare trauma	0.653
Incompetence	0.687
Caring family	0.720
Accidents to close person	0.593
Unstable caring environment	0.678
Safe environment	0.517
Lack of sexual/extreme trauma	0.650

### 3.3 Correlations between TAQ and other Maltreatment related scales or psychiatric scales

The total score of TAQ showed a significant relationship with other maltreatment scales and with psychiatric scales (Table 3). The correlation between age period TAQ score correlation and other maltreatment scales (MACE and VAQ) generally showed good correlations. The correlation coefficient value between MACE and age period for 0–6 years was 0.346, for 7–12 years was 0.596, and for 13–18 years was 0.704. Additionally, the correlation coefficient value between VAQ and age period for 0–6 years was 0.423, for 7–12 years was 0.535, and for 13–18 years was 0.612.

**TABLE 3:** Correlation between TAQ total or each period score and other scales

TAQ total score	Correlation coefficient
MACE(MULT)	0.562***
MACE(SUM)	0.677***
VAffectionQ(mother)	−0.216**
VAffectionQ(father)	−0.498***
VAQ(0−6 yr)	0.423***
VAQ(7−12 yr)	0.535***
VAQ(13−18 yr)	0.612***
STAI(Total)	0.363***
CES‐D	0.387***
Limbic system checklist	0.448***

Note

※ Only VAQ correlation analysis was done with TAQ score of each period, ※ There are two types of VAffectionQ responses: Mother and Father.

Abbreviations: CES‐D, Center for Epidemiologic Studies Depression Scale; MACE(MULT), Maltreatment and Abuse Chronology of Exposure Scale (Multiplicity); MACE(SUM), Maltreatment and Abuse Chronology of Exposure Scale (Summative); STAI, State‐Trait Anxiety Inventory; VAffectionQ, Verbal Affection Questionnaire; VAQ, Verbal Abuse Questionnaire.

** *p* < .01.

*** *p *< .001.

## 4 DISCUSSION

### 4.1 Binary coding

In the present study, we proposed 8 newly classified domains for the TAQ based on the application of binary scoring using 40 items. Participants who have severe traumatic experiences may not be adequately represented due to the study targeting the general population and not a clinical sample. Therefore, using the original TAQ scoring method, the conversion of the score “1” to “0” caused “0” inflation that is regarded as having no traumatic experience with the item (S3 file). Although scoring “1” indicates a mild experience, counting “1” makes sense to distinguish the presence or absence of an experience, not for the severity of the symptom. Therefore, it is more appropriate to score only “0” as “0” rather than including the converted score of “1” to “0.”

### 4.2 Simple scoring system

The item response theory has the advantage that each participant could be represented by a latent logit score (Morizot et al., 2009). It would have been best to construct individual scores using latent logit scores obtained for each domain if the number of items were sufficient. Therefore, it is recommended that there be at least five items in a domain. However, in the current TAQ, there are less than five items in four of the new domains (Accidents to close person, Unstable caring environment, Safe environment and Lack of sexual/extreme trauma). In these four domains, the latent logit score could not be obtained reliably using a Rasch model. This is also the reason why the number of “1's” (experience or not) was used in this study. If additional items are developed and a sufficient number of items per domain is produced in further studies, individual scoring based on the IRT would be possible.

### 4.3 Items having overlapped values in the same domain

The TAQ items 11 (My parents were divorced or separated) and 12 (I lived with different people at different times—like different relatives or foster families) in the “Unstable caring environment” domain had a similar nuance and item difficulty value. This may reflect similar content; therefore, it is necessary to consider deleting it or replacing it with other content when developing the item in future research. Conversely, the TAQ items 23 (People in my family were out of control) and 30 (I was beaten, kicked or punched by someone close to me) in the “Domestic violence” domain, the TAQ items 31 (I was in a situation in which I was convinced that I would be physically injured or lose my life) and 32 (Someone outside my family attacked me) in the “Safe environment” domain, and TAQ items 38 (Someone forced me to have sex against my will) and 40 (I believe that one of my brothers or sisters was sexually molested) in the “Sexual/ other rare trauma” domain also had overlapped values. However, it is valid to maintain these items because the object of measurement for each item is different.

### 4.4 The need to add new items and domains

TAQ items generally fit the Rasch model in each domain. The items of “Incompetence” did not, however, fit well in one domain (Table S3). In the case of the “Incompetence” domain, the items were simply a mixture of questions about individual achievement and care. Therefore, it was difficult to establish similar nuances for the items in this domain. Although the results of the factor analysis suggested that the items could be grouped into this domain because of the high level of connectivity between items, new item development may be beneficial to increase the stability of the “Incompetence” domain.

A trauma questionnaire, which includes all aspects of abuse, maltreatment, and neglect, could be very useful. Considering the “neglect” aspect, children could be neglected by caregivers or someone left in a miserable and unstable environment, such as war, could feel that they had been neglected. Therefore, both situations could be considered as a neglect in terms of an uncommon care environment. TAQ items 01 and 02 for neglect situations with caregivers are listed in the “Incompetence” domain. Apart from this, it is possible to check the situation in which the participant is in an unintended state, such as the “Unstable caring environment” and “Safe environment” domains. For these domains, it could be a TAQ‐specific advantage if these are supplemented more, such as developing suitable items. The TAQ has a lack of items for emotional maltreatment such as peer bullying. Given the increasing incidence and severity of bullying at school, there is a need to develop items that include bullying which is as serious as familial abuse (Kaess, 2018).

In the clinical situation, TAQ can be used as an efficient alternative measure of MACE due to the benefits of concise number of items and evaluation period. We also applied a binary scoring system, but since it maintains a scale from 0 to 3, previously acquired TAQ data can be rescored and used to retest the scoring system in various clinical populations. A Voxel‐Based Morphometry study with subjects exposed to occupational trauma reported a significant lower gray matter density in the left posterior cingulate in patients who developed Post‐Traumatic Stress Disorder as compared to ones who did not, and the negative relationship of the density with TAQ score (Nardo et al., 2010). In an explorative study for childhood experiences across developmental periods in psychiatric patients with different diagnoses such as alcohol‐related disorders, schizophrenia, affective disorders, and personality disorders, negative events measured with TAQ were reported in the patients with alcohol‐related and personality disorders more often than the ones with schizophrenia and affective disorders (Saleptsi et al., 2004). Thus, a reliable and valid assessment of the trauma experience of TAQ using the binary scoring system is expected to be useful in exploring its association with brain development or mental vulnerability in future studies. The significant correlation with depression scores suggest that the TAQ scores may also be associated with the psychological state of the participant at the time of assessment (Ben‐Zeev et al., 2009). Since the TAQ is a retrospective assessment, the depression score might be the result of being affected by the participant's current psychological state. Thus, it can also be inferred that past traumatic experiences could affect current state of mind. More specific research is needed to untangle this correlation.

## 5 CONCLUSION

The revised version of the Traumatic Antecedents Questionnaire presented in this study, based on participants' responses using Item Response Theory, is expected to facilitate fast screening of a large number of people because of its reasonable number of questions and various types of trauma domains. By using IRT, we were able to evaluate the distribution of the severity of items in each domain and identify the type of items that should be added. This version of the TAQ has good test–retest reliability and a significant correlation with other trauma and psychiatric scales; thus, it is expected that the measure could be more widely used if complemented by revised or new items in specific domains.

## CONFLICT OF INTEREST

The authors declare that they have no known competing financial interests or personal relationships that could have appeared to influence the work reported in this paper.

## AUTHOR CONTRIBUTIONS

G.S. devised the project, the main conceptual ideas and proof outline. K.P. collected the subjects' responses and analyzed the data. K.P. and G.S. carried out the implementation. B.J. performed the calculations and validated the results. K.P. and B.J. wrote the manuscript with input from all authors.

### Peer Review

The peer review history for this article is available at https://publons.com/publon/10.1002/brb3.1870.

## Supporting information

File S1

File S2

File S3

File S4

## Data Availability

Data can be available on the reasonable request.
