# Supplementary material for: Validation of the Traumatic Antecedents Questionnaire using item response theory
Source: Brain Behav. 2020 Oct 1;10(12):e01870. doi: 10.1002/brb3.1870 (PMC7749547; doi:10.1002/brb3.1870)
Supplement: Supplementary file 1 — File S1 [file BRB3-10-e01870-s001.docx]

**Supplemental results**

**S1. Distribution of age**

**
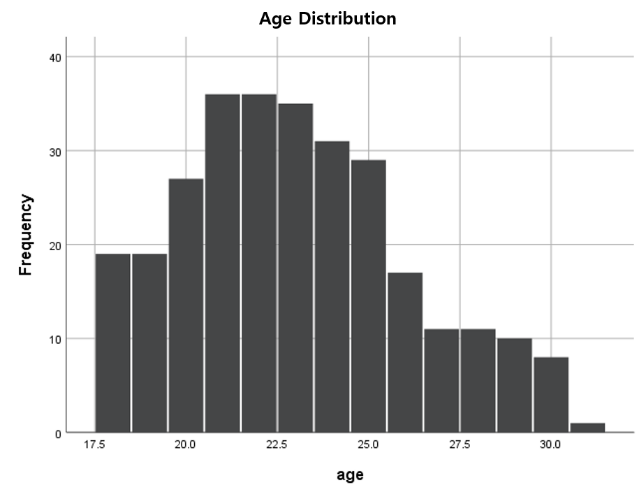
**

**S2. Frequency of responses in each item**

|  | **Frequency (%)** | | | | |  |  | **Frequency (%)** | | | | |
| --- | --- | --- | --- | --- | --- | --- | --- | --- | --- | --- | --- | --- |
| **Item** | **0** | **1** | **2** | **3** | **missing** |  | **Item** | **0** | **1** | **2** | **3** | **missing** |
| **1** | 77.6 | 13.5 | 4.2 | 1.5 | 3.3 |  | **22** | 96.9 | 1.6 | 1.0 | 0.3 | 0.1 |
| **2** | 81.8 | 7.2 | 3.1 | 5.1 | 2.8 |  | **23** | 85.7 | 7.5 | 2.2 | 3.2 | 1.4 |
| **3** | 66.1 | 19.3 | 6.7 | 3.6 | 4.4 |  | **25** | 77.8 | 12.6 | 4.8 | 3.8 | 1.0 |
| **4** | 62.9 | 21.9 | 8.9 | 2.2 | 4.2 |  | **26** | 94.8 | 1.8 | 1.5 | 1.3 | 0.7 |
| **5** | 58.1 | 18.8 | 10.3 | 9.1 | 3.7 |  | **27** | 89.1 | 4.4 | 2.6 | 3.2 | 0.7 |
| **6** | 72.1 | 11.0 | 8.7 | 5.4 | 2.8 |  | **28** | 97.7 | 0.9 | 0.7 | 0.5 | 0.1 |
| **7** | 87.1 | 4.9 | 3.7 | 2.0 | 2.2 |  | **29** | 95.2 | 1.9 | 1.6 | 1.0 | 0.3 |
| **8** | 25.1 | 19.1 | 14.1 | 29.9 | 11.8 |  | **30** | 85.7 | 9.1 | 2.8 | 1.7 | 0.7 |
| **10** | 82.7 | 6.7 | 4.5 | 2.5 | 3.6 |  | **31** | 90.9 | 5.0 | 2.5 | 0.7 | 1.0 |
| **11** | 92.6 | 1.6 | 1.6 | 3.8 | 0.4 |  | **32** | 90.7 | 5.8 | 2.1 | 0.8 | 0.7 |
| **12** | 92.3 | 4.8 | 1.7 | 0.9 | 0.3 |  | **33** | 94.4 | 4.6 | 0.5 | 0.1 | 0.4 |
| **13** | 83.9 | 13.0 | 2.0 | 0.4 | 0.7 |  | **34** | 94.9 | 4.4 | 0.4 | 0.1 | 0.1 |
| **14** | 90.7 | 6.6 | 1.4 | 1.0 | 0.3 |  | **35** | 98.1 | 0.9 | 0.5 | 0.3 | 0.1 |
| **15** | 80.3 | 13.3 | 3.9 | 1.9 | 0.6 |  | **36** | 94.2 | 3.7 | 1.0 | 0.6 | 0.4 |
| **16** | 94.6 | 3.0 | 1.0 | 0.5 | 0.9 |  | **37** | 95.1 | 3.3 | 0.8 | 0.2 | 0.5 |
| **17** | 83.6 | 7.3 | 4.8 | 2.1 | 2.2 |  | **38** | 98.9 | 0.5 | 0.3 | 0.1 | 0.2 |
| **18** | 82.7 | 9.3 | 3.8 | 2.9 | 1.3 |  | **39** | 99.2 | 0.2 | 0.4 | 0 | 0.1 |
| **19** | 72.9 | 12.8 | 5.5 | 5.3 | 3.4 |  | **40** | 98.4 | 0.5 | 0.4 | 0.1 | 0.6 |
| **20** | 70.6 | 14.9 | 6.6 | 5.2 | 2.8 |  | **41** | 90.5 | 6.2 | 1.3 | 1.3 | 0.6 |
| **21** | 82.6 | 9.0 | 4.3 | 2.6 | 98.5 |  | **42** | 97.2 | 1.3 | 0.5 | 0.3 | 0.7 |

* Item 9 and 24 were excluded because 42 items were used before the TAQ revision at the time of survey.

**S3. Results of Rasch analyses of each domain**

**S3.1 Domestic violence**

Table S1. Rasch analysis of Domestic violence items

|  | **Item fit Statistics** | **Wald test** |
| --- | --- | --- |

|  | | χ^2^ | df | p-value | Item difficulty | Outfit MSQ | | Infit  MSQ | Outfit t | Infit t |  | z-statistic | p-value |
| --- | --- | --- | --- | --- | --- | --- | --- | --- | --- | --- | --- | --- | --- |
| **TAQ 18** | 405.689 | | 424 | 0.731 | 0.209 | | 0.955 | 0.980 | -0.54 | -0.33 |  | -1.860 | 0.063 |
| **TAQ 19** | 510.650 | | 424 | 0.002 | -0.613 | | 1.202 | 1.154 | 2.90 | 3.12 |  | -0.502 | 0.616 |
| **TAQ 20** | 403.612 | | 424 | 0.754 | -0.911 | | 0.950 | 0.993 | -0.70 | -0.14 |  | -1.034 | 0.301 |
| **TAQ 21** | 437.537 | | 424 | 0.315 | 0.309 | | 1.029 | 1.013 | 0.37 | 0.23 |  | -0.038 | 0.970 |
| **TAQ 23** | 364.835 | | 424 | 0.983 | 0.660 | | 0.858 | 0.898 | -1.37 | -1.56 |  | 0.413 | 0.680 |
| **TAQ 25** | 338.396 | | 424 | 0.999 | -0.244 | | 0.796 | 0.852 | -3.21 | -3.01 |  | 0.745 | 0.456 |
| **TAQ 30** | 377.333 | | 424 | 0.950 | 0.589 | | 0.888 | 0.965 | -1.12 | -0.52 |  | 2.003 | 0.045 |

This domain consisted of 7 items (Table 2). Table S1 presents the Rach analysis for the Domestic violence domain. The mean square fit statistics were acceptable and ranged from 0.796–1.202. Figure 2 shows the Item Characteristic Curves (ICC), Item Fit Curves (IFC) and Test Information Function for Domestic violence. This domain was most informative with logit scores around 0 which suggests an average latent trauma level .

This domain covered the latent trait with logit scores of -2.89 (the lowest person value) to 2.87 (the highest person value). The Andersen LR test was non-significant (8.059, df = 6, p = 0.234) indicating acceptable fit. The Cronbach's alpha value was 0.837 that indicate consistency of these 7 items. Infit t-statistic values should conventionally be within the range of -1.96 to +1.96. T-values of 5 items were within the range except TAQ items 19 and 25. In homogeneity check revealed that TAQ item 30 had a p-value of 0.045 in the standard Wald test and was placed outside of the p-value line of the graphical model check (Figure 2). There was reasonable evidence of convergent validity. TAQ domestic violence scores correlated with the MACE Parental Verbal Abuse (r = 0.596), Parental Nonverbal Emotional Abuse (r = 0.552), Parental Physical Maltreatment (r = 0.520), Witnessing Interparental Violence (r = 0.487), Witnessing Violence to Siblings (r = 0.439), and Peer Emotional Abuse scores (r = 0.432).

**S3.2 Sexual/other rare trauma**

Table S2. Rasch analysis of Sexual/other rare trauma items

|  | **Item fit Statistics** | **Wald test** |
| --- | --- | --- |

|  | | χ^2^ | df | p-value | Item difficulty | Outfit MSQ | | Infit  MSQ | Outfit t | Infit t |  | z-statistic | p-value |
| --- | --- | --- | --- | --- | --- | --- | --- | --- | --- | --- | --- | --- | --- |
| **TAQ 14** | 152.248 | | 147 | 0.366 | -1.925 | | 1.029 | 1.037 | 0.76 | 1.09 |  | 0.763 | 0.445 |
| **TAQ 34** | 142.041 | | 147 | 0.600 | -1.303 | | 0.960 | 0.959 | -0.50 | -0.52 |  | -1.175 | 0.24 |
| **TAQ 35** | 103.198 | | 147 | 0.998 | 0.139 | | 0.697 | 0.736 | -1.37 | -1.30 |  | 1.532 | 0.126 |
| **TAQ 38** | 112.035 | | 147 | 0.986 | 0.849 | | 0.757 | 0.782 | -0.63 | -0.64 |  | -1.089 | 0.276 |
| **TAQ 39** | 138.693 | | 147 | 0.675 | 1.551 | | 0.937 | 0.767 | 0.05 | -0.39 |  | -0.794 | 0.427 |
| **TAQ 40** | 126.882 | | 147 | 0.883 | 0.849 | | 0.857 | 0.803 | -0.31 | -0.56 |  | 0.694 | 0.488 |
| **TAQ 42** | 126.914 | | 147 | 0.883 | 0.589 | | 0.888 | 0.965 | -1.12 | -0.52 |  | 0.713 | 0.476 |

This domain consisted of 7 items. Table S2 presents the Rach analysis for the Sexual/other rare trauma domain. The mean square fit statistics were in the acceptable range from 0.697-1.037. TAQ item 35 had an outfit mean square value < 0.7 suggesting a trend-level trait of overfitting.


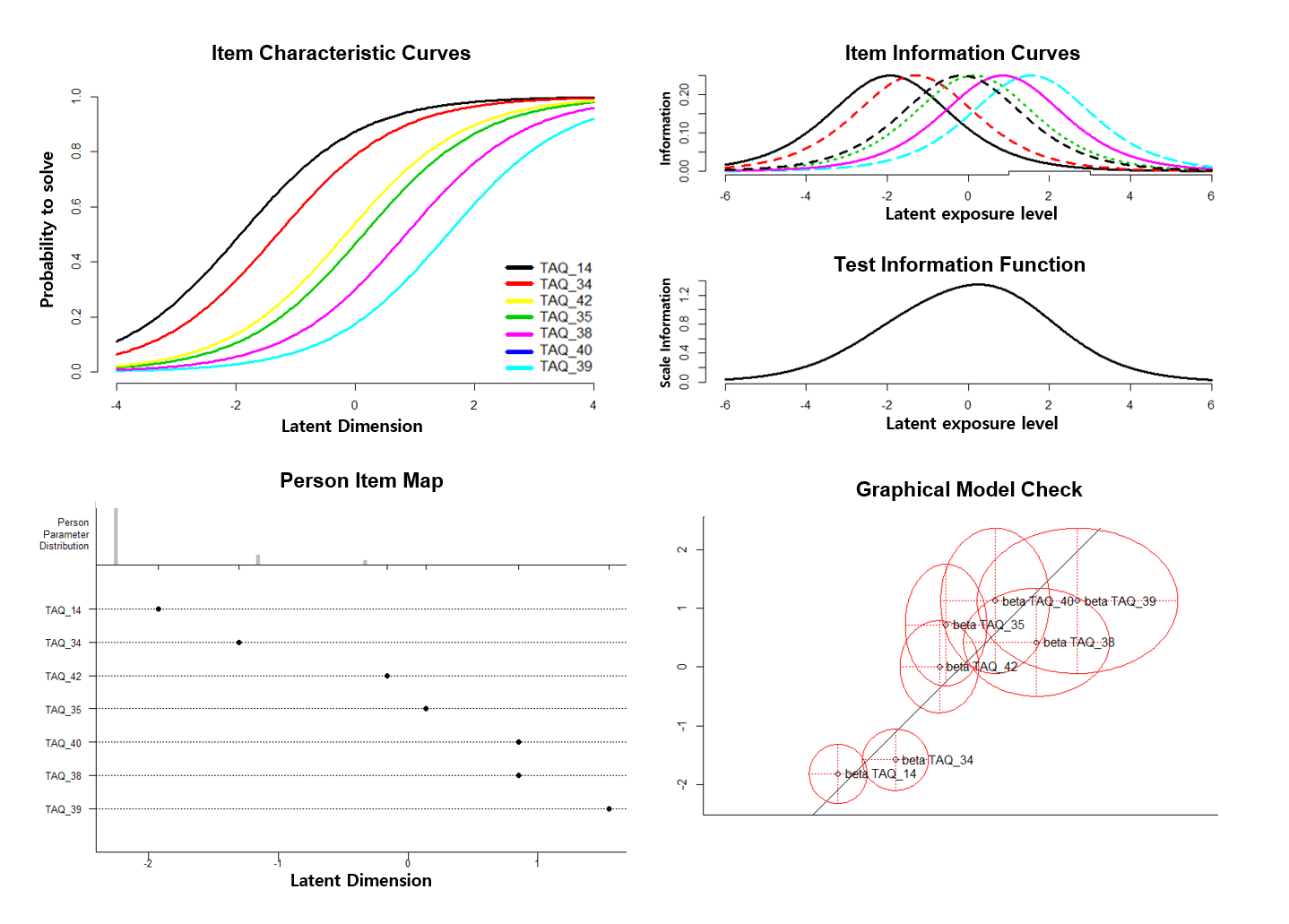


Figure S1. Results of the Rasch analysis of Sexual/Other rare trauma

Figure S1 shows the ICC, IFC and Test Information Function for Sexual/other rare trauma. This domain was most informative between logit scores of 0 and 1 which suggests a slightly higher than average latent trauma level. This domain covered the latent trait from logit scores of -2.25 to -0.33. The Andersen LR test was non-significant (8.044, df = 6, p = 0.235) indicating acceptable fit.

The Cronbach’s alpha value was 0.579 but increased to 0.637 when TAQ item 14 was removed. However, all 7 items, including TAQ 14, fit well based on the infit t-statistics and did not show any inhomogeneity with the Wald test and the graphical model check.

**S3.3 Incompetence**

Table S3. Rasch analysis of Incompetence items

|  | **Item fit Statistics** | **Wald test** |
| --- | --- | --- |

|  | χ^2^ | df | p-value | Item difficulty | Outfit MSQ | Infit  MSQ | Outfit t | Infit t |  | z-statistic | p-value |
| --- | --- | --- | --- | --- | --- | --- | --- | --- | --- | --- | --- |
| **TAQ 01** | 644.963 | 845 | 1 | 0.730 | 0.762 | 0.848 | -2.89 | -3.23 |  | 0.338 | 0.735 |
| **TAQ 02** | 984.725 | 845 | 0.001 | 1.027 | 1.164 | 0.927 | 1.52 | -1.35 |  | 3.617 | < 0.001 |
| **TAQ 03** | 719.789 | 845 | 0.999 | 0.019 | 0.851 | 0.933 | -2.58 | -1.74 |  | 0.737 | 0.461 |
| **TAQ 04** | 691.231 | 845 | 1 | -0.258 | 0.817 | 0.877 | -3.58 | -3.46 |  | -3.480 | 0.001 |
| **TAQ 05** | 770.559 | 845 | 0.968 | -0.611 | 0.911 | 0.953 | -1.78 | -1.33 |  | -1.404 | 0.160 |
| **TAQ 08** | 751.061 | 845 | 0.991 | -2.193 | 0.888 | 0.934 | -1.13 | -1.68 |  | -1.830 | 0.067 |
| **TAQ 17** | 648.006 | 845 | 1 | 1.287 | 0.766 | 0.985 | -2.06 | -0.22 |  | 1.092 | 0.275 |

This domain of 7 items had 6 reversed scored items (TAQ 01, 02, 03, 04, 05, 08). Table S3 presents the Rach analysis for the Incompetence domain. The mean square infit and outfit statistics were acceptable as they were all between 0.762 - 1.164.


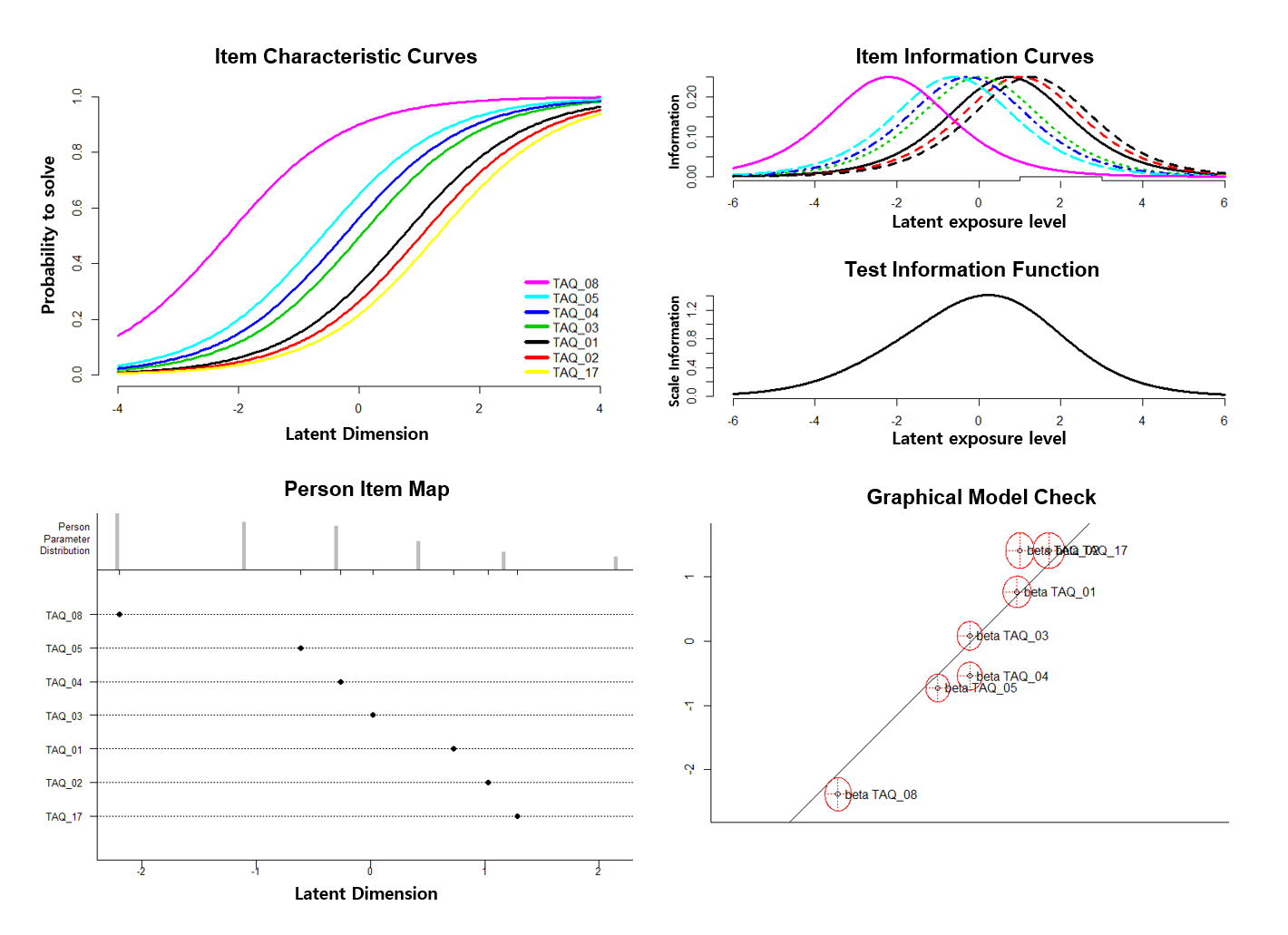


Figure S2. Results of the Rasch analysis of Incompetence

This domain was most informative between logit scores of 0 and 1 which suggests a slightly higher than average latent trauma level. This domain covered the latent trait with logit scores from -3.40 to 3.19. In this domain, the Andersen LR test had significant p-value (25.986, df = 6, p < 0.001) suggesting that individuals at the same level of an underlying trait in the “Incompetence” domain may respond to a specific item depending on age. Logit score in the older group (23 years or older), compared to the younger, was low for TAQ items 01, 02, and 03 while high in TAQ items 04, 05, 08, and 17. Although the Cronbach's alpha value was 0.639, fit t-values of 4 items (TAQ 01, 03, 04, and 17) were out of the range from -1.96 to +1.96. Further, based on the Wald test, TAQ items 02 and 04 had a significant p-value under 0.05 and were also located outside of the line at the graphical model check (Figure S2).

**S3.4 Caring family**

Table S4. Rasch analysis of Caring family items

|  | **Item fit Statistics** | **Wald test** |
| --- | --- | --- |

|  | | χ^2^ | df | p-value | Item difficulty | Outfit MSQ | | Infit  MSQ | Outfit t | Infit t |  | z-statistic | p-value |
| --- | --- | --- | --- | --- | --- | --- | --- | --- | --- | --- | --- | --- | --- |
| **TAQ 06** | 300.892 | | 312 | 0.664 | -2.351 | | 0.961 | 0.913 | -0.24 | -2.18 |  | -0.887 | 0.375 |
| **TAQ 22** | 163.391 | | 312 | 1 | 1.703 | | 0.522 | 0.819 | -1.88 | -1.10 |  | -1.610 | 0.107 |
| **TAQ 26** | 218.594 | | 312 | 1 | 0.507 | | 0.698 | 0.803 | -2.17 | -1.72 |  | 1.039 | 0.299 |
| **TAQ 27** | 223.172 | | 312 | 1 | -0.639 | | 0.713 | 0.757 | -3.83 | -3.87 |  | -0.051 | 0.959 |
| **TAQ 29** | 107.313 | | 312 | 1 | 0.781 | | 0.343 | 0.554 | -5.00 | -4.02 |  | 1.566 | 0.117 |

This scale consisted of 5 items. Table S4 presents the Rach analysis for the Caring family domain. The mean square infit and outfit statistics of these items were acceptable, but 3 items had mean square fit values < 0.7 indicating some degree of overfit or item redundancy.


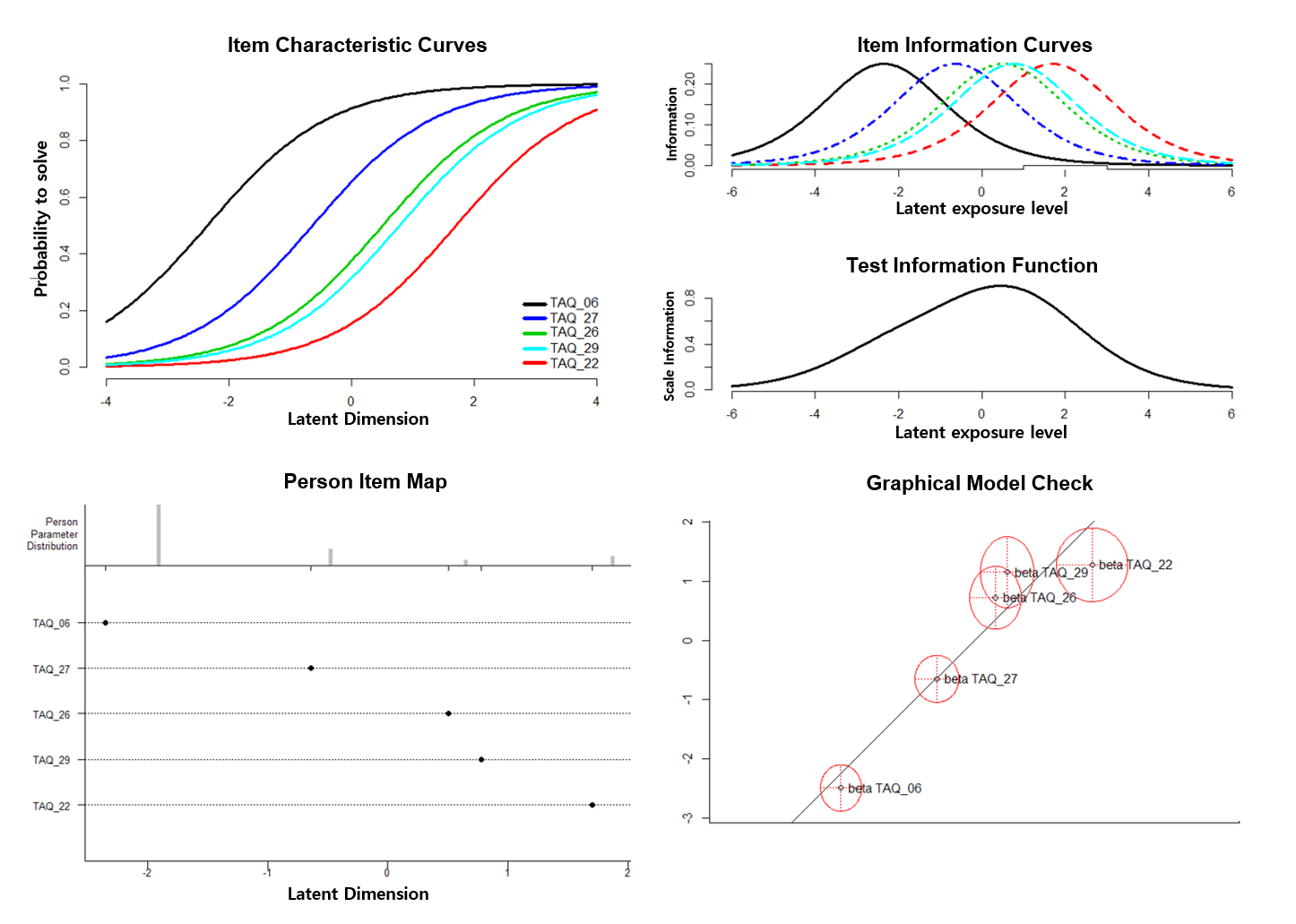


Figure S3. Results of the Rasch analysis of Caring family

This domain was most informative between logit scores of 0 and 1 which suggests a slightly higher than average latent trauma level. This domain covered the latent trait from logit scores of -3.42 to 3.14. The Andersen LR test was non-significant (5.929, df = 4, p = 0.205) indicating acceptable fit.

The Cronbach’s alpha value was 0.618. If the TAQ item 06 was eliminated, the value increased to 0.752. However, item 06 was only associated with other items in this domain. These 5 items had homogeneity based on good fit Wald test indices and the graphical model check (Figure S3). The TAQ Caring family domain scores correlated with the MACE Parental Nonverbal Emotional Abuse (r = 0.407) and Witnessing Interparental Violence (r = 0.443) domain scores.

**S3.5 Accidents to close person**

Table S5. Rasch analysis of Accidents to close person items

|  | **Item fit Statistics** | **Wald test** |
| --- | --- | --- |

|  | | χ^2^ | df | p-value | Item difficulty | Outfit MSQ | | Infit  MSQ | Outfit t | Infit t |  | z-statistic | p-value |
| --- | --- | --- | --- | --- | --- | --- | --- | --- | --- | --- | --- | --- | --- |
| **TAQ 13** | 253.779 | | 267 | 0.710 | -0.450 | | 0.947 | 0.956 | -1.14 | -1.11 |  | 1.025 | 0.305 |
| **TAQ 15** | 245.412 | | 267 | 0.824 | -0.879 | | 0.916 | 0.949 | -1.48 | -1.49 |  | -1.286 | 0.198 |
| **TAQ 16** | 169.008 | | 267 | 1 | 1.329 | | 0.631 | 0.700 | -2.96 | -3.16 |  | 0.187 | 0.852 |

This scale consisted of 3 items. Table S5 presents the Rach analysis for the Accidents to close person domain. Mean square infit and outfit statistics of these 3 items were ranged from 0.631 - 0.956. Item 16 had an outfit mean square fit < 0.7, which suggests some degree of overfitting.


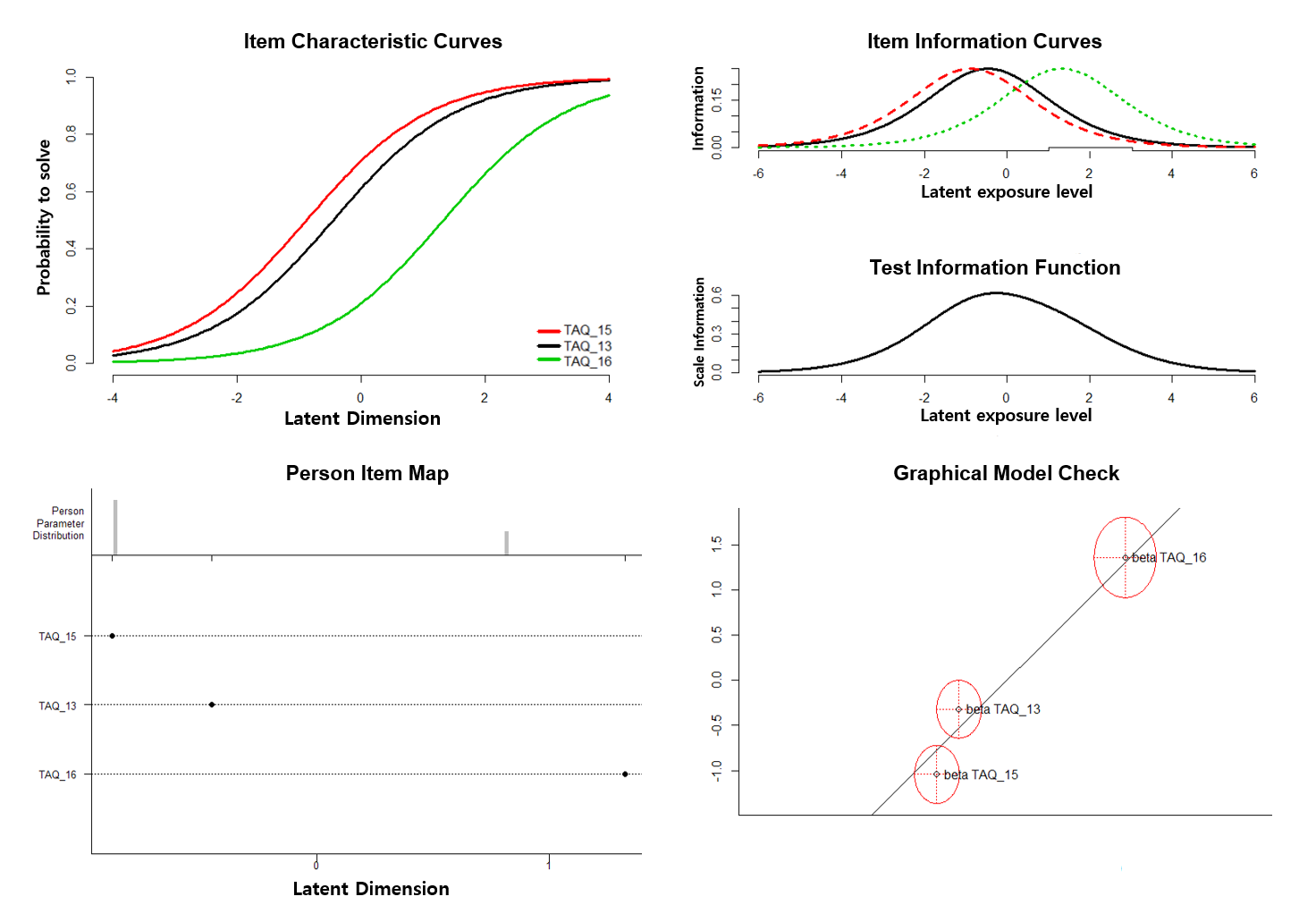


Figure S4. Results of the Rasch analysis of Accidents to close person

This domain was most informative between logit scores of -1 and 0 which suggests a slightly lower than average latent trauma level. This domain covered the latent trait logit scores from -0.87 to 0.82. The Andersen LR test was non-significant (2.655, df = 2, p = 0.265) indicating acceptable fit.

The Cronbach's alpha value of this domain was 0.514 indicating consistency. The homogeneity check suggests all items in this domain has acceptable fit (Figure S4).

**S3.6 Unstable caring environment**

Table S6. Rasch analysis of Unstable caring environment items

|  | **Item fit Statistics** | **Wald test** |
| --- | --- | --- |

|  | | χ^2^ | df | p-value | Item difficulty | Outfit MSQ | | Infit  MSQ | Outfit t | Infit t |  | z-statistic | p-value |
| --- | --- | --- | --- | --- | --- | --- | --- | --- | --- | --- | --- | --- | --- |
| **TAQ 07** | 278.178 | | 261 | 0.222 | -0.171 | | 1.062 | 1.052 | 1.00 | 0.9 |  | -0.923 | 0.356 |
| **TAQ 10** | 272.251 | | 261 | 0.303 | -0.731 | | 1.039 | 1.047 | 0.77 | 1.27 |  | 0.505 | 0.614 |
| **TAQ 11** | 230.014 | | 261 | 0.917 | 0.462 | | 0.878 | 0.883 | -1.27 | -1.39 |  | -1.305 | 0.192 |
| **TAQ 12** | 209.696 | | 261 | 0.991 | 0.441 | | 0.800 | 0.829 | -2.19 | -2.11 |  | 1.631 | 0.103 |

This scale consisted of 4 items. Table S6 presents the Rach analysis for the Unstable caring environment domain. The mean square infit and outfit statistics were acceptable as they were all between 0.8 - 1.062.


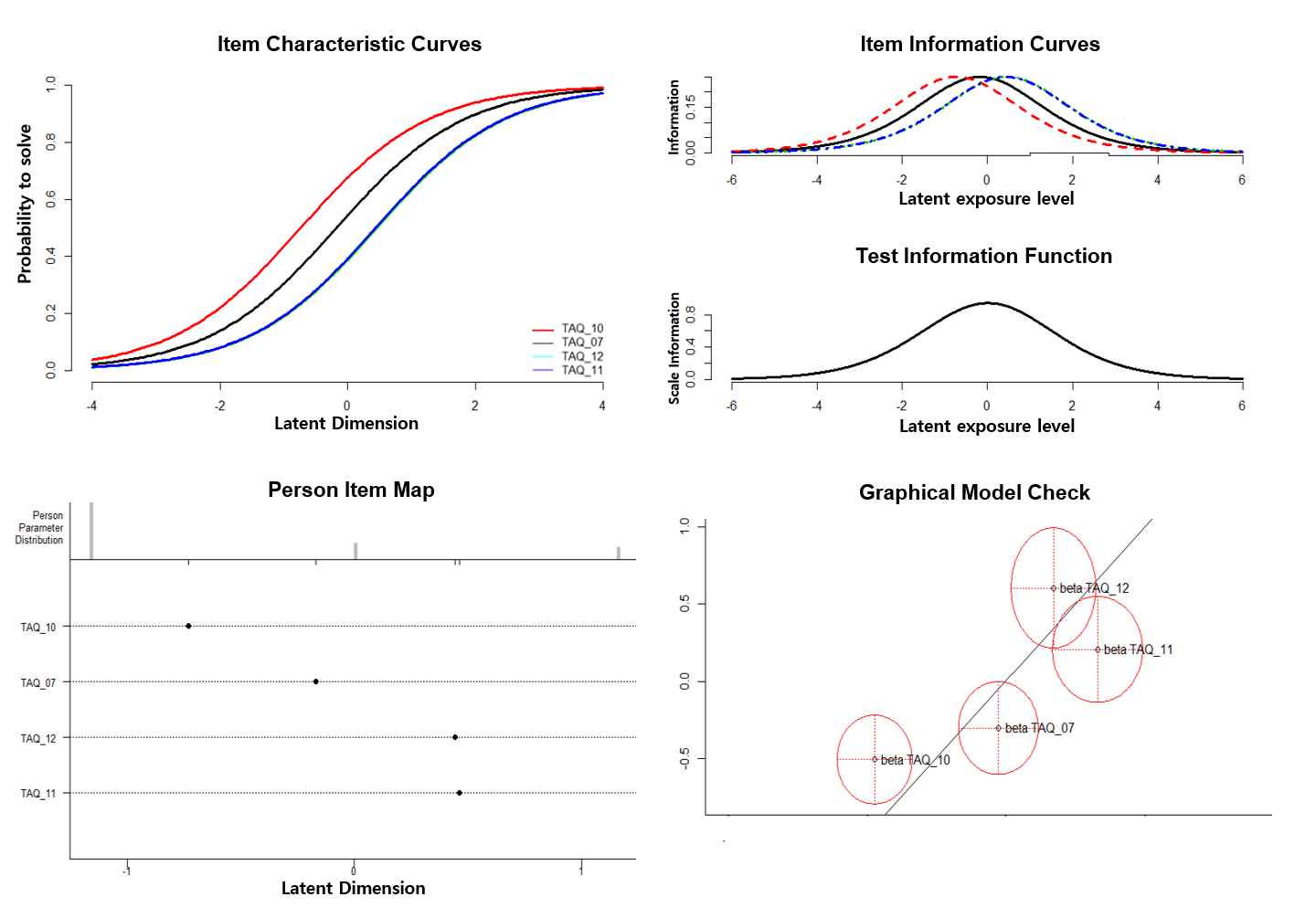


Figure S5. Results of the Rasch analysis of Unstable caring environment

This domain was most informative at logit scores around 0 which suggests an average latent trauma level. This domain covered the latent trait from logit scores of -1.22 (the lowest person value) to 1.23 (the highest person value). The Andersen LR test was non-significant (4.054, df = 3, p = 0.256) indicating acceptable fit. Cronbach’s alpha value of this scale was 0.516 and all items had good fit indices for infit t-statistics, Wald test, and the graphical model check (Figure S5). This domain correlated with the MACE Parental Nonverbal Emotional Abuse (r = 0.421) domain scores.

**S3.7 Safe environment**

Table S7. Rasch analysis of Safe environment items

|  | **Item fit Statistics** | **Wald test** |
| --- | --- | --- |

|  | | χ^2^ | df | p-value | Item difficulty | Outfit MSQ | | Infit  MSQ | Outfit t | Infit t |  | z-statistic | p-value |
| --- | --- | --- | --- | --- | --- | --- | --- | --- | --- | --- | --- | --- | --- |
| **TAQ 28** | 170.221 | | 174 | 0.567 | 1.361 | | 0.973 | 0.871 | -0.04 | -0.68 |  | -1.026 | 0.305 |
| **TAQ 31** | 169.575 | | 174 | 0.581 | -0.564 | | 0.969 | 0.953 | -0.50 | -0.86 |  | 0.181 | 0.857 |
| **TAQ 32** | 160.02 | | 174 | 0.769 | -0.699 | | 0.914 | 0.934 | -1.52 | -1.38 |  | 1.677 | 0.093 |
| **TAQ 33** | 176.052 | | 174 | 0.442 | -0.099 | | 1.006 | 1.010 | 0.10 | 0.16 |  | -0.101 | 0.920 |

This scale consisted of 4 items. Table S7 presents the Rach analysis for the Safe environment domain. All item mean square statistics were acceptable.


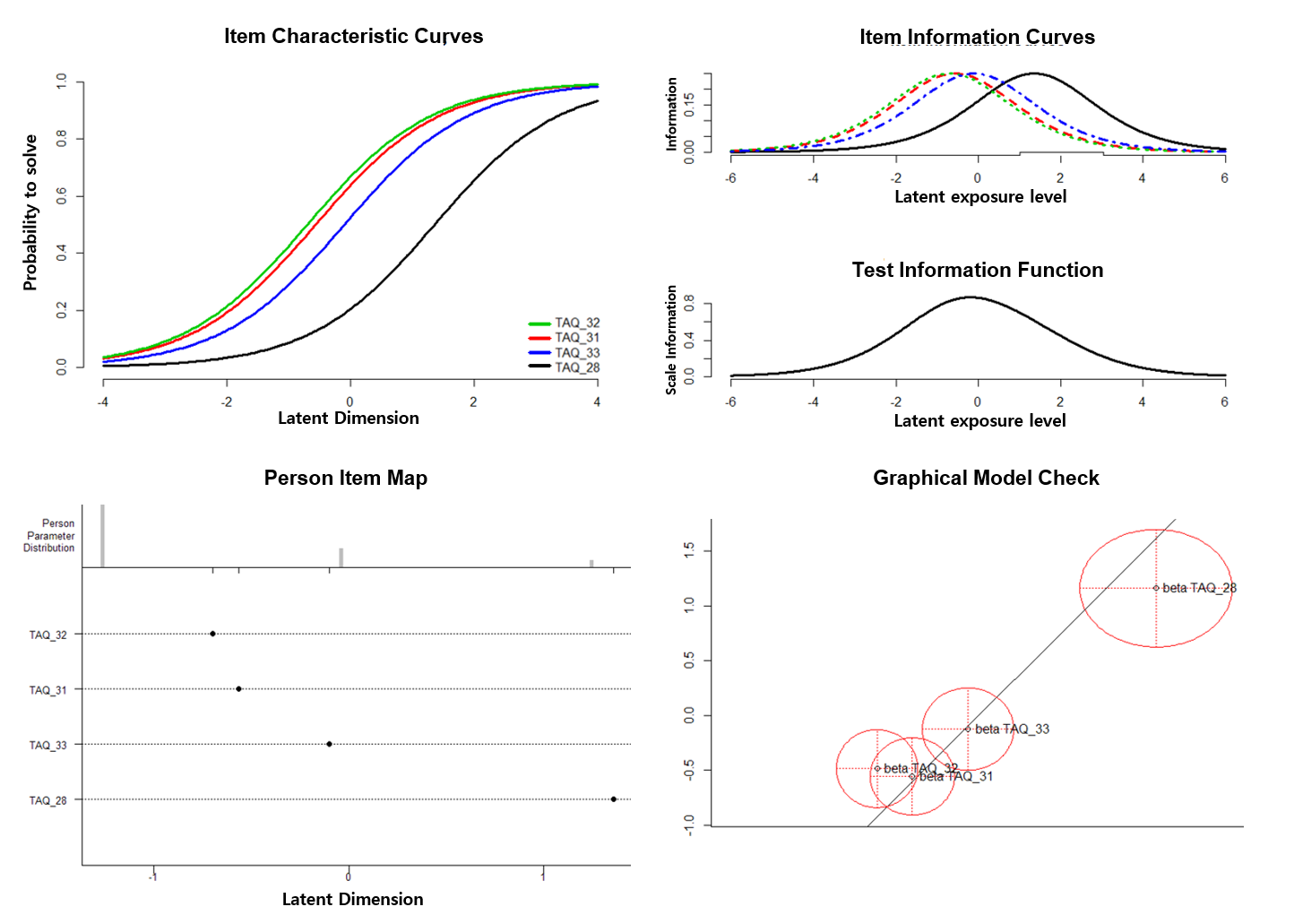


Figure S6. Result of Rasch analysis of Safe environment

This domain was most informative between logit scores of -1 and 0 which suggests a slightly lower than the average latent trauma level. This domain covered the latent trait logit scores of -1.26 to 1.25. The Andersen LR test was non-significant (2.884, df = 3, p = 0.41) indicating acceptable fit.

The Cronbach’s alpha value was 0.582 and infit t-statistics for all items were in an acceptable range. Wald test p-values and the graphical model check showed good homogeneity (Figure S6). This domain’s scores correlated with the MACE Peer Physical Bullying (r = 0.439) domain scores.

**S3.8 Lack of sexual/extreme trauma**

Table S8. Rasch analysis of Lack of sexual/extreme trauma items

|  | **Item fit Statistics** | **Wald test** |
| --- | --- | --- |

|  | | χ^2^ | df | p-value | Item difficulty | Outfit MSQ | | Infit  MSQ | Outfit t | Infit t |  | z-statistic | p-value |
| --- | --- | --- | --- | --- | --- | --- | --- | --- | --- | --- | --- | --- | --- |
| **TAQ 36** | 122.32 | | 130 | 0.672 | -0.033 | | 0.934 | 0.934 | -1.07 | -1.07 |  | 1.276 | 0.202 |
| **TAQ 37** | 120.251 | | 130 | 0.719 | 0.407 | | 0.918 | 0.925 | -0.91 | -0.88 |  | -1.361 | 0.174 |
| **TAQ 41** | 140.253 | | 130 | 0.254 | -0.374 | | 1.071 | 1.067 | 1.25 | 1.35 |  | 0.150 | 0.881 |

This scale consisted of three items. Table S8 presents the Rach analysis for the Lack of sexual/extreme trauma domain. The mean square fit statistics were acceptable and ranged from 0.918 – 1.071.


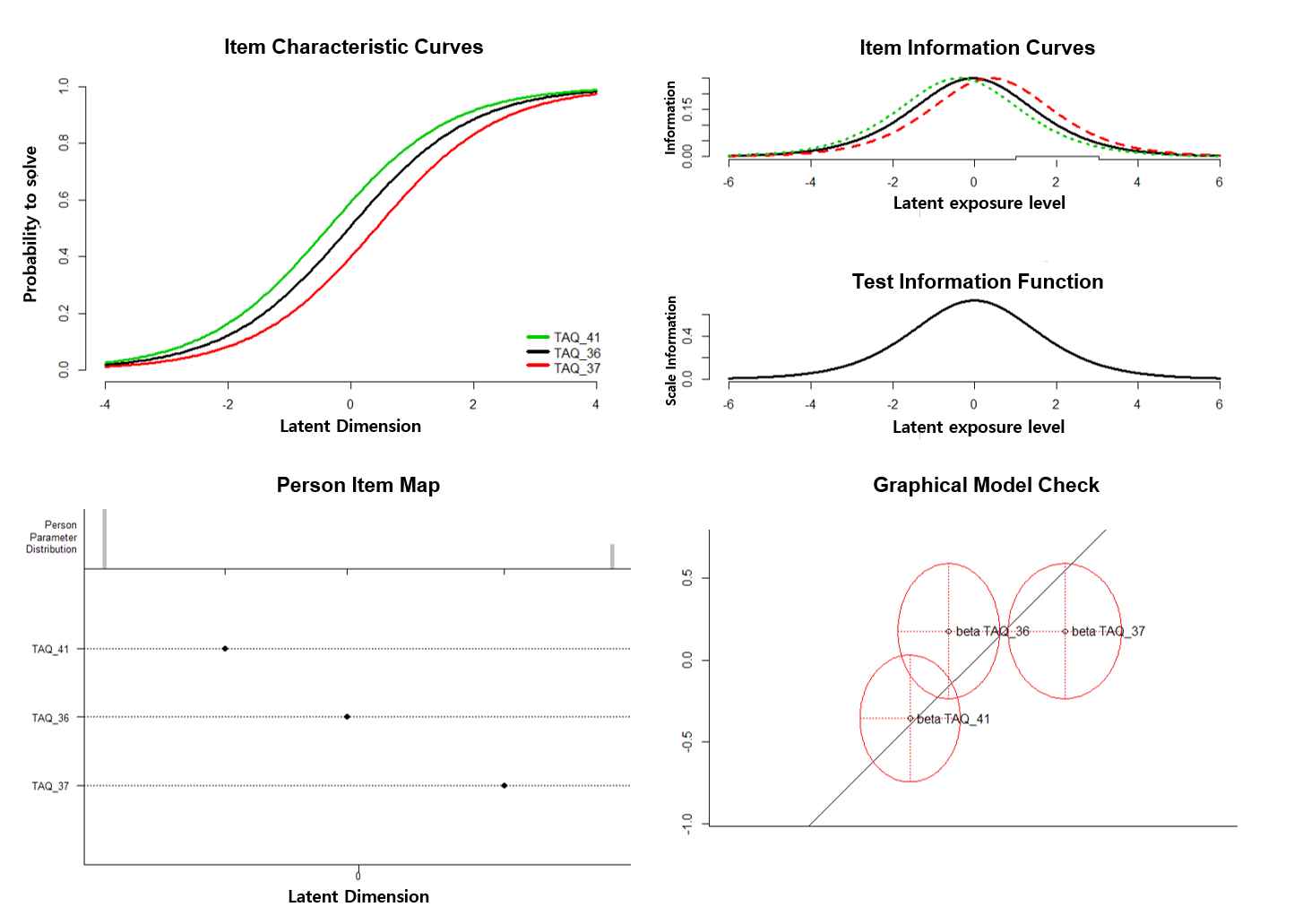


Figure S7. Result of Rasch analysis of Lack of sexual/extreme trauma

This domain was most informative with logit scores around 0 which suggests an average latent trauma level. This domain covered the latent trait from logit scores of -0.71 (the lowest person value) to 0.71 (the highest person value). The Andersen LR test was non-significant (2.24, df = 2, p = 0.326) indicating acceptable fit. The Cronbach’s alpha value was 0.458. Wald test p-values and the graphical model check also suggested good homogeneity (Figure S7). This domain’s scores correlated with the MACE Sexual Abuse (r = 0.423) domain scores.

**S3.9 Items showing poor internal consistency**

Internal consistency was checked to group items that may express one index (Table 1). TAQ item 06 (Somebody in my family had so many problems that there was little left for me) was included in the “Caring family” domain, but when excluding this item, the Cronbach's alpha coefficient of the domain increased from 0.693 to 0.752. Likewise, excluding TAQ item 14 (I had a serious illness and/or had to be hospitalized for a medical problem) increased the Cronbach's alpha of the “Sexual / other rare trauma” domain from 0.579 to 0.637. This means that both items have less contextual similarity with other questions in the same domain. The participant’s response results to TAQ items 06 and 14 had a different pattern from other items in each domain. If a participant answered TAQ item 06 or 14 positively, there were cases where the participant did not positively answered other items in the same domain. This pattern could have caused the internal consistency to decrease. However, based on factor analysis, TAQ items 06 and 14 were considered to be much more similar in the current domain than others.

S4. Linear mixed model

S4.1 Comparison of gender differences in each developmental period

The TAQ has the potential capacity to delineate the developmental time course of exposure to maltreatment. In the present study, using the MASS package (version 7.3-51.4), generalized linear mixed effect models were performed to ascertain: (1) whether there were significant differences in severity of recollected exposure for the 8 domains and total score across developmental stages; (2) to indicate whether levels of exposure were influenced by gender.

S4.2 Differences in recalling traumatic experience by gender and age periods

Mixed model analysis was performed to determine how the total score and scores of each domain were related to age period and gender factors (Table S9). A significant main effect of gender indicated that males had higher scores in the “Incompetence” and “Unstable caring environment” domains. On the other hand, “Sexual/rare trauma” and “Lack of sexual/extreme trauma” domains showed higher scores for females. The significant main effect of age periods suggested that all domains showed higher scores in one or two periods after age 0-6.

The total score showed significant gender by age period interactions. Females had a significantly different distribution of scores in Total score, Domestic violence, Incompetence, Accidents to close person, Unstable caring environment, and Lack of sexual/extreme trauma domains. Sexual/rare trauma and Safe environment scores for females, compared with males, had significantly changed differential age period scores at the 13-18-year period. There was no gender by period interaction for the “Caring family” domain.

It is generally known that males are exposed to physical violence at higher rates than females, while females are subjected to higher rates of sexual assault than males at a young age (Trends, 2016). However, there were no gender differences in domains related to physical abuse such as “Domestic violence” and “Safe environment” in this study. The domains related to sexual abuse, “Sexual/rare trauma” and “Lack of sexual/extreme trauma,” were found to have significantly different trends for females. However, because these domains do not focus solely on physical violence, it is possible that gender differences were less noticeable than in previous studies.

Table S9. Linear mixed analyses of TAQ age periods and gender

|  |  | **Total** | **Domestic violence** | **Sexual/rare trauma** | **Caring family** | **Accidents to close person** | **Unstable caring environment** | **Safe environment** | **Lack of sexual/extreme trauma** | **Incompetence** |
| --- | --- | --- | --- | --- | --- | --- | --- | --- | --- | --- |
|  |  | Correlation coefficients (SE) | | | | | | | | |
| **Fixed part** |  |  |  |  |  |  |  |  |  |  |
|  | intercept | 2.126(0.060)^***^ | 1.552(0.112)^***^ | 0.357(0.094)^***^ | 0.849(0.108)^***^ | 0.819(0.106)^***^ | 1.572(0.115)^***^ | -0.090(0.114) | -0.302(0.115)^**^ | 3.178(0.064)^***^ |
|  | Gender | -0.125(0.069) | -0.129(0.082) | 0.282(0.111)^*^ | 0.040(0.100) | 0.144(0.078) | -1.043(0.094)^***^ | 0.267(0.126) | 0.802(0.120)^***^ | -0.221(0.048)^***^ |
|  | Period(7-12yr) | 0.427(0.033)^***^ | 0.450(0.033)^***^ | 0.300(0.055)^***^ | 0.570(0.043)^***^ | 1.161(0.035)^***^ | 0.457(0.034)^***^ | 1.326(0.055)^***^ | 0.791(0.059)^***^ | 0.082(0.020)^***^ |
|  | Period(13-18yr) | 0.278(0.034)^***^ | 0.536(0.033)^***^ | 0.122(0.057)^*^ | 0.360(0.045)^***^ | 0.603(0.038)^***^ | 0.239(0.036)^***^ | 0.897(0.058)^***^ | 0.676(0.060)^***^ | 0.067(0.020)^**^ |
|  | Gender X Period(7-12) | 0.162(0.047)^***^ | 0.092(0.044)^***^ | -0.168(0.088) | -0.039(0.058) | 0.337(0.055)^***^ | 0.331(0.051)^***^ | 0.027(0.079) | 0.228(0.077)^**^ | 0.138(0.030)^***^ |
|  | Gender X Period(13-18) | 0.151(0.048)^**^ | 0.150(0.043)^*^ | 0.199(0.088)^*^ | -0.076(0.061) | 0.216(0.060)^***^ | 0.291(0.053)^***^ | 0.177(0.082)^*^ | 0.573(0.077)^***^ | 0.067(0.030)^*^ |
|  |  |  |  |  |  |  |  |  |  |  |
| **Random part** | Participant | 0.557(0.746) | 2.732(1.653) | 1.124(1.060) | 2.123(1.457) | 2.383(1.544) | 2.749(1.658) | 1.909(1.382) | 1.937(1.392) | 0.9008(0.950) |
| **Deviance** |  | 2474.4 | 5947.2 | 2952.5 | 3673.6 | 11162.3 | 5193.9 | 4676.7 | 5091.5 | 6253.3 |
| **AIC** |  | 2488.4 | 5961.2 | 2966.5 | 3687.6 | 11176.3 | 5207.9 | 4690.7 | 5105.5 | 6267.3 |

^*^p<0.05, ^**^p<0.01, ^***^p<.001; SE: Standard Error
